# Tuberculosis cutis orificialis with perianal involvement

**DOI:** 10.1590/0037-8682-0145-2023

**Published:** 2023-07-24

**Authors:** Gabriel Castro Tavares, Nurimar Conceição Fernandes

**Affiliations:** 1 Universidade Federal do Rio de Janeiro, Hospital Universitário Clementino Fraga Filho, Serviço de Dermatologia, Rio de Janeiro, RJ, Brasil. Universidade Federal do Rio de Janeiro Hospital Universitário Clementino Fraga Filho Serviço de Dermatologia Rio de Janeiro RJ Brasil

A 25-year-old man, a lifelong resident of Rio de Janeiro, presented with a 3-month history of a painful ulcer in the perianal region, fatigue, and evening fever. He had no history of cough, weight loss, altered bowel habits, or other comorbidities, or any relevant family history. He was a smoker with a history of high alcohol intake. Physical examination (Figure 1) revealed a tender fusiform ulcer in the perianal region, 2 cm in diameter, with erythematous indurated borders and a clean and friable base, without signs of inflammation or satellite lymph node enlargement. A spindle cutaneous biopsy was performed of the center and border of the ulcer for histopathological analysis, which revealed only dermal fibrosis. HIV, and hepatitis B and C serology, and quantitative VDRL were negative. Chest computed tomography showed an infiltrate with a characteristic “tree-in-bud” pattern (Figure 2) and the diagnosis of pulmonary tuberculosis was confirmed by sputum culture. Ulcer tissue tested positive for *Mycobacterium tuberculosis* on GeneXpert and culture, despite the lack of alcohol-acid bacilli. The patient was diagnosed with perianal cutaneous tuberculosis concomitant with pulmonary tuberculosis. He was treated with rifampicin, isoniazid, pyrazinamide, and ethambutol for 2 months; followed by rifampin and isoniazid for the following 4 months. By the end of the second month, the perianal lesion had healed (Figure 3). This report describes a rare form of cutaneous tuberculosis in an immunocompetent young man, and illustrates the importance of molecular methods (GeneXpert) for making the diagnosis^1^^,^^2^^,^^3^.

**FIGURE 1:**
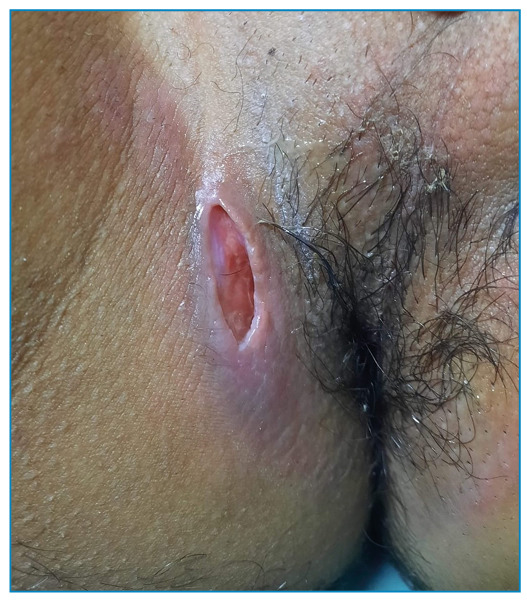
Perianal ulcer.

**FIGURE 2:**
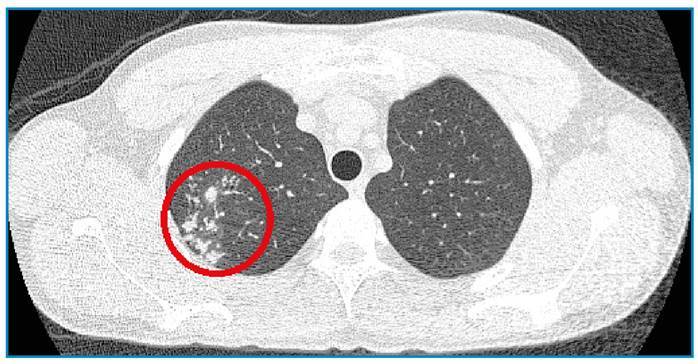
Chest tomography: “tree-in-bud pattern” (red circle).

**FIGURE 3:**
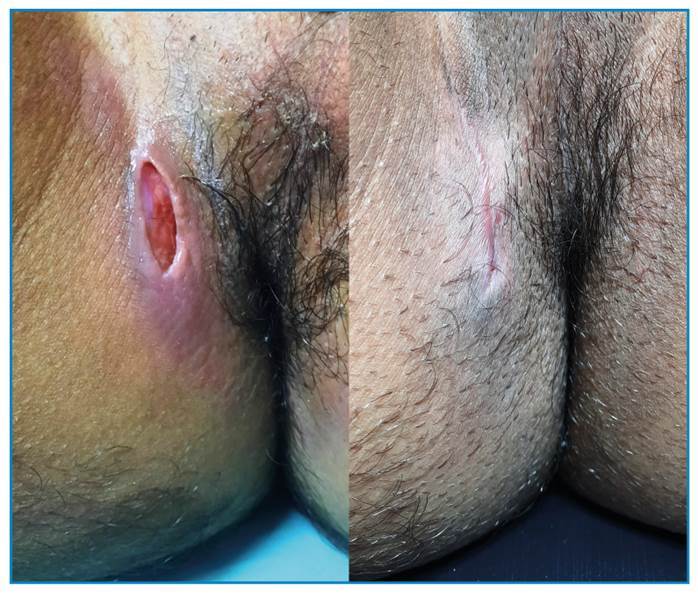
Perianal ulcer before (left) and after (right) 2 months of treatment.

## References

[1] 1. Brito AC, Oliveira CMM, Unger DA, Bittencourt MJS. Cutaneous tuberculosis: epidemiological, clinical, diagnostic and therapeutic update. An Bras Dermatol. 2022;97(2):129-44. 10.1016/j.abd.2021.07.004 PMC9073256 34996655

[2] 2. Dey B, Deshpande AH, Bhat SH, Singh A. Tuberculosis cutis orificialis with underlying pulmonary tuberculosis in an immunocompetent man. J Lab Physicians. 2018;10(4):457-9. 10.4103/JLP.JLP_4_18 PMC6210836 30498322

[3] 3. Lima T, Belotti N, Nardi S, Pedro H. Teste rápido molecular GeneXpert MTB/RIF para diagnóstico da tuberculose. Revista Pan-Amazônica de Saúde. 2017;8(2):65-76.

